# Mir-29c Expression in Glioma and Its Effects on Tumor Cell Proliferation and Apoptosis

**Published:** 2020-02

**Authors:** Peiquan HUI, Yuling WANG, Bing CHEN, Zengwu WANG, Shiqiang QIN

**Affiliations:** 1. Department of Neurosurgery, People's Hospital of Weifang, Weifang, Shandong, China; 2. Department of Ultrasonography, People's Hospital of Weifang, Weifang, Shandong, China

**Keywords:** MicroRNAs, Glioma, Expression level, Cell proliferation, Apoptosis

## Abstract

**Background:**

To investigate the expression of microRNA-29c (miR-29c) in glioma and its effects on cell proliferation and apoptosis.

**Methods:**

A retrospective analysis was performed on 76 glioma patients in People's Hospital of Weifang, Weifang, Shandong, China from May 2013 to June 2017 (experimental group) and 63 healthy subjects in the same period (control group). qRT-PCR was used to detect the miR-29c expression. Changes of serum miR-29c expression level and the correlation of miR-29c of glioma patients with the degree of tumor differentiation and pathological type were observed. Cells were grouped before transfection into blank group (no transfection), negative control group (transfected with miRNA NC) and experimental group (transfected with miR-29c mimics). CCK-8 assay was used to detect cell proliferation, flow cytometry to detect apoptosis.

**Results:**

Expression of miR-29c in serum was significantly lower in experimental group than that in control group (*P*<0.05). The expression level of miR-29c of glioma patients increased with the degree of tumor differentiation (*P*<0.05). miR-29c in serum was not significantly correlated with the pathological type.

**Conclusion:**

miR-29c could inhibit the proliferation of glioma cells and promote apoptosis. miR-29c is lowly expressed in glioma, and the overexpression of which in glioma cells can inhibit tumor cells proliferation and promote apoptosis. It may be a tumor suppressor miRNA of glioma, and the expression level of which can be used as reference for evaluating the grade of glioma. It is indicated that the abnormal expression of miR-29c may be a key factor in the occurrence and development of glioma.

## Introduction

Glioma is a common intracranial tumor whose incidence is the first in intracranial tumors (1). At present, it is mainly treated by surgical resection in clinical practice, combined with radiotherapy and chemotherapy for comprehensive treatment. Nevertheless, its high recurrence rate and mortality make its treatment effects and prognosis poor (2). In addition, the growth of glia is usually infiltrated, so conventional imaging examination can not accurately evaluate the condition (3). In recent years, with the development of molecular biology, a large number of studies have found that miRNAs can regulate cells’ biological functions such as proliferation and apoptosis by regulating the expression of genes (4). People are beginning to realize that finding an effective molecular gene has important clinical significance for the early diagnosis and treatment of glioma patients (5). Overexpression of Gαi protein and RTK in gliomas formed the complex, and transducted Akt-mTOR signaling, which may be one of the reasons for promoting the growth of glioma U8MG cells (6).

As a member of the miR-29s family (7), miR-29c was first discovered in nasopharyngeal carcinoma. Its expression is significantly lower in nasopharyngeal carcinoma tissues than that in normal tissues, and it is considered to be closely related to the metastasis and invasion of nasopharyngeal carcinoma (8). The low expression of miR-29c is an important factor in the occurrence and development of some malignant tumors (9). For example, closely related to the development of leukemia and liver cancer, which is considered to be a marker for judging tumor prognosis (10, 11). In recent years, some scholars have begun to study the expression of miR-29a in glioma (12), and some studies have explored the mechanism of the role of microRNAs in glioma, such as microRNAs-92b (13) and microRNAs-130b (14) regulate the proliferation, migration, invasion and apoptosis of glioma cells through PTEN/Akt signaling pathway.

Therefore, in this study, the microRNA-29c (miR-29c) expression in glioma and its effects on cells’ biological functions such as proliferation were investigated, in order to provide a new molecular direction for the diagnosis and treatment of glioma.

## Methods

### General information

A retrospective analysis was performed on 76 glioma patients treated in People's Hospital of Weifang, China from May 2013 to June 2017 as experimental group, including 37 males and 39 females, with an average age of (41.2±4.6) years old. They were divided into grade I–II with 41 cases and grade III–IV with 35 cases based on the degree of tumor differentiation, who were also divided into 27 astrocytoma patients, 25 glioblastoma multiforme patients and 24 mixed glioma patients based on pathological types. And 63 healthy subjects in the same period were selected as control group. There was no significant difference in gender, age and BMI between the two groups of subjects (*P*>0.05).

Inclusion criteria: patients diagnosed as glioma by pathological diagnosis; subjects confirmed to be healthy by physical examination were included in control group. Exclusion criteria: patients with other severe organ or other tumor diseases were excluded; patients with communication and mental disorders; patients who did not cooperate to conduct the experiment.

All subjects and their families agreed to participate in the experiment and signed an informed consent form. This experiment has been approved by the Ethics Committee of People's Hospital of Weifang (Table 1).

**Table 1:** General information sheet

***Variable***	***Experimental group (n=76)***	***Control group (n=63)***	***t/X^2^***	**P**
Gender			0.016	0.900
Male	37(48.68)	30(47.62)		
Female	39(51.32)	33(52.38)		
Age (yr)			0.034	0.854
≥40	47(61.84)	38(60.32)		
<40	29(38.16)	25(39.68)		
BMI			0.854	0.334
≥23	41(53.95)	33(52.38)		
<23	35(46.05)	30(47.62)		
Degree of differentiation				
Grade I∼II	41(53.95)	-	-	-
Grade III∼IV	35(46.05)	-	-	-
Pathological types				
Astrocytoma	27(35.53)	-	-	-
Glioblastoma multiforme	25(32.89)	-	-	-
Mixed glioma	24(31.58)	-	-	-
Coagulation function				
APTT (s)	28.89±2.71	29.02±2.78	0.278	0.781
PT (s)	11.97±1.02	12.05±0.99	0.467	0.462
FIB (g/l)	3.14±0.23	3.17±0.22	0.781	0.436
TT (s)	14.78±1.62	14.55±1.59	0.840	0.402
Smoking			0.004	0.951
Yes	39(51.32)	32(50.79)		
No	37(48.68)	31(49.21)		
Liver function Index				
Total serum protein (g/L)	71.67±2.54	71.91±2.67	0.542	0.589
Glutamic-pyruvic transaminase (μmol/L)	26.13±4.27	26.19±4.08	0.084	0.933
Total bilirubin (μmol/L)	11.25±2.08	11.13±2.11	0.336	0.737
Renal function Index (μmol/L)				
Creatinine	69.45±4.23	70.12±4.19	0.934	0.352
Serum urea	5.78±0.79	5.71±0.87	0.497	0.620
Uric acid	297.56±13.73	295.09±14.01	1.046	1.297

### Experimental materials and reagents

Unknown human glioblastoma cell U87MG was purchased from Shanghai Zeye Biotechnology Co., Ltd. (Cat. No. AC319), real-time quantitative PCR instrument from American BioRad Company, flow cytometer CytoFLEX LX from American Beckman Company, DMEM medium from American Gibco Company, fetal bovine serum (FBS) and trypsin from American Hyclone Company, Trizol reagent from American Applide Invitrogen Company, qPCR kit and minScript reverse transcription kit from Dalian TaKaRa Company. Primers for miR-29c mimics, miRNA NC and internal reference U6 were designed and synthesized by Shanghai Jima Company. CCK-8 reagent was purchased from American Promega Company, Annexin V-FITC/PI apoptosis kit from Jiangsu Kaiji Biotechnology Co., Ltd.

### Experimental methods

#### Detection of serum miR-29c expression in patients by RT-PCR

Three ml of venous blood was extracted from all subjects on an empty stomach in the morning, and then centrifuged at 3000 r/min for 5 min. After that, the serum was placed in a refrigerator at −80°C for testing. Trizol reagent (15) was added to the serum reagent tube to extract total RNA. An ultraviolet spectrophotometer was used to detect RNA purity and concentration. One μg of total RNA was taken to synthesize cDNA according to the reverse transcription kit instructions. The OD260/OD280 ratio is 1.8, and the OD260/OD230 ratio is 2.2. 2 μl of the synthesized cDNA was taken to perform qPCR. Reaction conditions were: pre-denaturation at 95 °C for 3 min, and then at 95 °C for 15 s, at 60 °C for 45 s, for a total of 40 cycles. U6 as an internal reference, the expression of miR-29c was detected. Primer sequences are shown in Table 2. 2^−ΔCT^ was used to express the relative expression of genes. The experiment was repeated 3 times.

**Table 2:** Related primer sequence listing

**Primer**	**Upstream primer**	**Downstream primer**
miR-29c	5′-ACACTCCAGCTGGGTGACCGATTTCTCCTC-3′	5′-TGGTGTCGTG GAGTCG-3′
U6	5′-CTCGCTTCGGCAGCACA-3′	5′-AACGCTTCACGAATTTGCGT-3′

#### Cell culture, passage and transfection

Human glioma cell U87MG was taken out and placed in a medium containing 10% fetal bovine serum, cultured in an incubator at 37 °C and 5% CO_2_. When cells’ adherent growth reached 80%, they were washed with PBS, digested with 25% trypsin, and then continuously cultured for passage in the 10% solution at 37 °C and 5% CO_2_. Cells grown in log phase were selected for transfection after grouping. Cells with no transfection were set as blank group, those transfected with miRNA NC as negative control group, and those transfected with miR-29c mimics (16) as experimental group. Specific transfection steps: first, cells were seeded in a 6-well plate at a density of 3×10^5^ cells/well. Then, lipofectamine 2000 and DNA were diluted and mixed according to the instructions of lipofectamine 2000 transfection kit, placed at room temperature for 5 min. Next, the mixture was mixed with cells, transfected at 37 °C and 5% CO_2_. After transfection for 48 h, PCR technique was used to detect the expression of miR-29c in U87MG cells transfected with miR-29c and miR-NC. And cells were collected for subsequent experiments.

#### CCK-8 detection of cell proliferation

Cells were collected at 48 h after transfection, diluted to 3×10^4^ cells/ml, and seeded in a 96-well plate. One hundred μl of cells was inserted into each well, cultured at 37 °C and 5% CO_2_. 10 μl of CCK8 solution was added to each well on the 1st, 2nd, 3rd, 4th, and 5th days after cells’ adherent growth, respectively. After that, the reagent was added, and cells were continuously cultured in the incubator. After culture for 2 h, a microplate reader was used to measure the OD value at 450 nm for detecting cell proliferation and plotting the growth curve. The experiment was repeated 3 times.

#### Flow cytometry detection of apoptosis (17)

Annexin V-FITC/PI double staining combined with flow cytometry was used to detect apoptosis. U87MG cells transfected with miR-29c and miRNA NC were seeded in a 6-well plate at a density of 3×10^5^ cells/well. After incubation for 24 h, they were washed twice with PBS. Then, 5 μl of Annexin V-FITC was added, reaction at room temperature for 10 min. After that, 10 μl of PI was added and incubated at room temperature for 20 min in the dark. Finally, flow cytometer was used to detect apoptosis. The experiment was repeated 3 times.

### Statistical methods

In this study, SPSS18.0 software (Boyi Zhixun (Beijing) Information Technology Co., Ltd.) was used for the statistical analysis of data, GraphPad Prism 6 software for plotting all the pictures in this experiment. The chi-square test was used for the comparison of count data. Measurement data were expressed as mean ± standard deviation. The *t* test was used for analysis between the two groups, analysis of variance for comparison among multiple groups. When *P*<0.05, there is a statistically significant difference.

## Results

### miR-29c expression in serum of two groups

The relative expression of miR-29c in serum of patients was (0.39±0.07) in experimental group, significantly lower than (1.12±0.18) in control group, with a statistically significant difference (*P*<0.05). In experimental group of patients, the lower the degree of tumor differentiation was, the lower the relative expression of miR-29c was (*P*<0.05). There was no significant difference in the relative expression of miR-29c among different pathological types (Table 3).

**Table 3:** miR-29c expression in serum of patients with different pathological types and different degrees of tumor differentiation

	**Degree of differentiation**		**Pathological types**		
	*Grade I∼II*	*Grade III∼IV*	*Astrocytoma*	*Glioblastoma multiforme*	*Mixed glioma*
n	41	35	27	25	24
miR-29c	0.31 ±0.04	0.45 ±0.08	0.38 ±0.08	0.40 ±0.06	0.39 ±0.09

### miR-29c expression in three groups of cells after transfection

PCR was used to detect the miR-29c expression in cells after transfection. The relative expression of miR-29c was (14.32±4.57) in experimental group, significantly higher than that in blank group and control group, with a statistically significant difference (*P*<0.05) (Table 4).

**Table 4:** miR-29c expression in U87MG cells after transfection for 48h

***Factor***	***Experimental group***	***Blank group***	***Negative control group***	***t***	**P**
miR-29c	14.32±4.57	0.40 ±0.09	0.39 ±0.05	27.84	<0.001

### Effects of miR-29c on cell proliferation

**Fig. 1:**
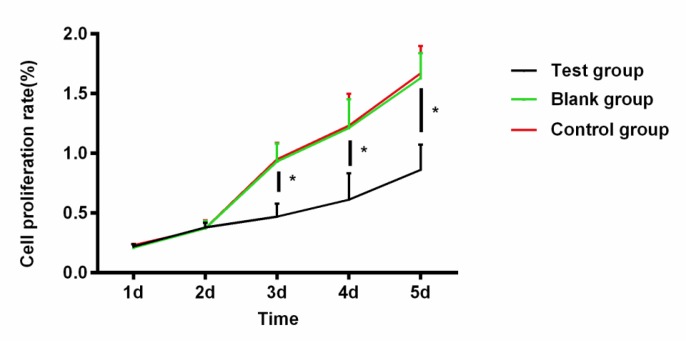
Effects of miR-29c on cell proliferation There was no significant difference in the proliferation ability among three groups of cells on the 1st and 2nd days (*P*>0.05). That of cells in experimental group began to be significantly lower than that in blank group and negative control group from the 3rd day, with a statistically significant difference (*P*<0.05). Note: ^*^ indicates that *P*<0.05

The detection of cell proliferation at different time points after transfection showed that there was no significant difference in the proliferation ability of cells among three groups on the 1st and 2nd days. That of cells in experimental group began to be significantly lower than that in blank group and negative control group from the 3rd day, with a statistically significant difference (*P*<0.001) (Table 5, Fig. 1).

**Table 5:** Effects of miR-29c on cell proliferation

***Group***	***1d***	***2d***	***3d***	***4d***	***5d***
Experimental group	0.22±0.02	0.38±0.04	0.47±0.11	0.61±0.22	0.86±0.21
Blank group	0.21±0.03	0.37±0.06	0.93±0.15	1.21±0.24	1.63±0.21
Negative control group	0.23±0.01	0.37±0.07	0.95±0.14	1.23±0.27	1.67±0.23
*F*	0.643	0.030	12.24	6.245	13.29
*P*	0.559	0.971	<0.050	<0.050	<0.050

### Effects of miR-29c on apoptosis

The apoptosis rate was (3.49±0.21) % in control group, (3.12±0.18) % in blank group and (7.21±0.15) % in experimental group. That was significantly higher in experimental group than that in negative control group blank group, with a statistically significant difference (*P*<0.001). There was no significant difference in that between negative control group and blank group.

## Discussion

Currently, more reliable bases can be provided for the diagnosis, prognostic prediction and development of treatment plans of glioma in the molecular layer (18, 19). Multiple miRNA are abnormally expressed in glioma (20). miR-137 has an effect on the proliferation and angiogenesis of glioma. The mechanism is caused by the role of the targeted gene EZH2 (21). miR-29c, one of the important members of the miR-29s family, has function as a tumor suppressor gene in various tumors. For instance, in lung cancer, it inhibits the expression of EMT induced by SP1/TGF-0205 through inhibiting the expression of SP1/TGF-0205 in vivo, thereby exerting a tumor suppressor effect (22). It has also been found to exert a tumor suppressor effect by targeting and DNA methylases 3a and 3b in nasopharyngeal carcinoma (23). At present, few studies are about the miR-29c expression in glioma and its effects on glioma cells, but miR-29c was involved in the chemosensitivity of glioma cells to temozolomide (24). Therefore, in this study, the miR-29c expression in glioma and its effects on tumor cell proliferation and apoptosis were investigated.

First, PCR was used to compare the miR-29c expression in serum of glioma patients and healthy subjects and the effects of different pathological factors on serum miR-29c. The results showed that the relative expression of miR-29c in serum of patients was significantly lower in experimental group than that in control group. In experimental group of patients, the lower the degree of tumor differentiation was, the lower the relative expression of miR-29c was. There was no significant difference in that among different pathological types.

The higher the malignant degree of glioma is, the lower the expression level of miR-29c is (25). The abnormally low expression of miR-29c is considered as a key molecule event in the occurrence and development of glioma. This study did not directly explore the expression of miR-29c in different degrees of differentiation of glioma, but it is consistent with our conclusions indirectly.

Then, in order to investigate the effects of miR-29c on the biological functions of glioma cells, glioma cell U87MG was cultured and transfected with miR-29c mimics. In U87MG cells transfected with miR-29c mimics, it was detected that the miR-29c content was significantly higher than that in blank group and negative control group. The transfection was confirmed to be successful and subsequent experiments were continued. In the following, the proliferation and apoptosis of the three groups of cells were compared. The results showed that there was no significant difference in the proliferation ability of cells among three groups on the 1st and 2nd days. That of cells in experimental group began to be significantly lower than that in blank group and negative control group from the 3rd day. The apoptosis rate was significantly lower in control group and blank group than that in experimental group. Having a significant inhibitory effect on cell proliferation, and the overexpression of miR-29c in glioma can trigger cell cycle arrest and inhibit angiogenesis (26), which is similar to our conclusions. In addition, the biological functions of glioma cell U251 transfected with miR-29c were studied (27). The results have shown that the proliferation and metastasis ability of cells transfected with miR-29c are significantly inhibited. It is suggested that miR-29c has a certain inhibitory effect on the differentiation and growth of glioma.

## Conclusion

MiR-29c is lowly expressed in glioma, and the overexpression of which in glioma cells can inhibit the proliferation of tumor cells and promote their apoptosis. However, in this study, the specific mechanism of miR-29c involved in the biological functions of glioma cells has not been explored. It is hoped that the majority of scholars can conduct further in-depth researches on this.

## Ethical considerations

Ethical issues (Including plagiarism, informed consent, misconduct, data fabrication and/or falsification, double publication and/or submission, redundancy, etc.) have been completely observed by the authors.
